# Training in obstetrics and gynecology between reality and vision: results of a JAGO–NOGGO survey in 601 physicians (NOGGO—Monitor-12 trial)

**DOI:** 10.1007/s00404-024-07508-z

**Published:** 2024-04-16

**Authors:** Gabriel von Waldenfels, Maximilian Heinz Beck, Janina Semmler, Annika Gerber, André Hennigs, Ruth Vochem, Jens-Uwe Blohmer, Barbara Schmalfeldt, Klaus Pietzner, Jalid Sehouli

**Affiliations:** 1 Department of Gynecology, Breast Center, Campus Mitte, Charité-Universitätsmedizin Berlin, Berlin, Germany; 2 Young Academy of Gynecologic Oncology (JAGO, ), Berlin, Germany; 3 Department of Gynecology, Center for Oncological Surgery, Campus Virchow Klinikum, Charité-Universitätsmedizin Berlin, Berlin, Germany; 4 Department of Obstetrics, Campus Virchow Klinikum, Charité-Universitätsmedizin Berlin, Berlin, Germany; 5 Fertility Doctors Berlin, Berlin, Germany; 6 Department of Obstetrics and Gynecology, University Hospital Heidelberg, Im Neuenheimer Feld 440, 69120 Heidelberg, Germany; 7 TFP Kinderwunsch Klagenfurt, Klagenfurt, Austria; 8 Department of Gynecology and Gynecologic Oncology, University Medical Center Hamburg-Eppendorf, Hamburg, Germany; 9 German Society of Gynecology and Obstetrics (DGGG), Berlin, Germany

**Keywords:** Education, Obstetrics and gynecology, Surgical training, Residency, OBGYN

## Abstract

**Purpose:**

The primary objective of this study was to establish a benchmark by collecting baseline data on surgical education in obstetrics and gynecology in Germany, including factual number of operations performed.

**Materials and methods:**

A nationwide anonymous survey was conducted in Germany between January 2019 and July 2019 utilizing a specially designed questionnaire which addressed both residents and senior trainers.

**Results:**

A total of 601 participants completed the survey, comprising 305 trainees and 296 trainers. The trainees reported performing a median of 125 non-obstetric surgeries (IQR: 41–332) and 75 obstetric procedures (IQR: 27–168) independently. While most last-year residents managed to meet the targeted numbers for minor surgical procedures outlined in the logbook, they fell short of achieving the required numbers for major operations, such as hysterectomies or more complex laparoscopies. Although both trainees and trainers emphasized the significance of surgical training, the overall quality of the training was rated poorly, particularly by trainees. This was attributed to a high proportion of administrative tasks and a deficiency in teaching time within the operating theater. External fellowship and mentoring programs, as well as the implementation of regular, centralized reviews of residency training, were identified as potentially beneficial by both trainees and trainers.

**Conclusion:**

The findings of this survey should serve as a wake-up call both within and outside of Germany, highlighting the importance of comprehensive and structured surgical training to enhance long-term patient care and increase satisfaction among obstetrics and gynecology trainees.

## What does this study add to the clinical work

**Table:** 

In this nationwide survey conducted in Germany among trainees and trainers in OBGYN, the assessment of surgical training quality revealed dissatisfaction, with unmet benchmarks for key surgical procedures.	

## Background

Quality of training is the backbone for medical knowledge transfer and ongoing professional development in clinical routine and residency programs. However, medical education faces significant challenges due to the exponential growth of medical knowledge and the resulting complexity of surgical and medical procedures. These challenges are further compounded by the broad spectrum of Obstetrics and Gynecology (OBGYN) and its subdisciplines, making it difficult to provide in-depth training to all residents in each area. Given that surgeries are a major component of clinical practice, the acquisition of surgical skills is a fundamental aspect of OBGYN training. Currently, the standard training model for gynecologic surgery involves integrating residents into surgical teams and providing guidance by experienced colleagues. However, this educational system is constantly challenged by cost pressures, limited resources in healthcare systems, high workloads in clinical routine, and individual factors. To standardize training, in Germany, the training of OBGYN residents follows a 5-year curriculum that is comparable to international practice [1–3]. This curriculum is structured by further training regulations from the German Medical Association (Bundesärztekammer), which includes a logbook to document particular surgical skills and a minimum number of surgical interventions. The current required numbers vary between the federal German states, but usually comprise about 100–200 minor gynecologic surgeries, 50–100 major gynecologic surgeries, and 25 operative interventions in obstetrics [2–4]. However, despite its importance, there is a lack of actual data on the current quality of training in clinical routine [5–7].

## Materials and methods

The primary objective of this study was to establish a benchmark by collecting baseline data on surgical education in OBGYN in Germany, including the factual number of operations performed. Secondary objectives were to identify challenges and opportunities for improvement in the field of surgical training in OBGYN and to gather diverse perspectives and appropriate suggestions from trainers and trainees in this area.

### Design and participants

This national anonymous online survey was conducted from 24.01.2019 to 10.07.2019 by the Young Academy of Gynecologic Oncology (Junge Akademie Gynäkologische Onkologie—JAGO) of the North-Eastern German Society of Gynecologic Oncology (Die Nord-Ostdeutsche Gesellschaft für Gynäkologische Onkologie—NOGGO), with support from the Working Group of Gynecologic Oncology of the German Society for Gynecology and Obstetrics and the German Cancer Society (Arbeitsgemeinschaft Gynäkologische Onkologie—AGO). The study was approved by the local ethics committee at the Charité University Hospital in Berlin (local reference EA1/042/23).

The survey was developed as part of a scientific and clinical fellowship program of the JAGO. During a five-part modular workshop, the fellows conducted a comprehensive literature review and developed a questionnaire under the advice of interprofessional and interdisciplinary experts. The survey was designed for both gynecologists in training and those responsible for training more junior colleagues. The questionnaire was distributed with the use of institutional mailing lists, such as those of the NOGGO and the newsletter of the Young Forum of the German Society for Gynecology and Obstetrics (DGGG). The data were collected via the web-based Surveymonkey^©^ software.

### Questionnaire

The questionnaire consisted of 30 items for trainees and 24 items for trainers, with the option to skip individual questions. It included 11 trainee-specific questions, 5 trainer-specific questions, and 19 questions that were common for both trainers and trainees. The quality and importance of surgical training were measured using rating scales, while the exact number of surgeries was collected through free-text responses. Dichotomous questions were used to determine whether there were difficulties with staffing or manipulation of logbook figures and whether annual training review meetings were held. Questions that allowed for multiple answers and free-text responses were used to evaluate current support, identify difficulties, and suggest improvements.

### Data analysis

Categorical variables are presented as numbers and percentages. Unless otherwise specified, continuous variables are presented as median and interquartile range. Comparisons between trainees and trainers were performed using an unpaired *t* test. A subgroup analysis was conducted for independently performed surgeries by residents in their last year of training (5th year). *P* values less than 0.05 were considered significant. The analysis was performed using Microsoft Excel 2022® (Microsoft Corp./USA) and Prism 9.0® (GraphPad Software, Inc./USA).

## Results

### Study population

A total of 601 participants completed the survey, with 305 trainees and 296 trainers. Participants from all 16 German federal states were represented. General characteristics of the participants are presented in Table 1. The majority of participants worked in maximum care or university hospitals, that were authorized for full residency training. Most of institutions were certified as gynecologic and breast oncologic centers. Only a minority worked in offices (Table 1). The majority of trainees were in their third to fifth year of training. Most of the trainers were consultants (38.18%, *n* = 105) or senior consultants (22.91%, *n* = 63), followed by chief physicians (20.73%, *n* = 57) and specialists (15.25%, *n* = 42). More than half of the trainers (58.03%, *n* = 172) had one or more subspecialties, including Gynecological Oncology (42.34%, *n* = 116), Fetal Medicine (23.72%, *n* = 65), or Gynecological Endocrinology and Reproductive Medicine (5.11%, *n* = 14).

**Table 1:** General characteristics of the participants

		Trainees	Trainers			Trainees	Trainers
**Number**		305 (100)	296 (100)			**Intended**	**Acquired**
**Sex**	*Female*	234 (86.99)	147 (53.26)	**Specialization**	*Gynaecologic oncology*	118 (43.38)	116 (42.34)
	*Male*	35 (13.01)	129 (46.74)		*Fetal medicine*	62 (22.79)	65 (23.72)
**Age** (years, average ± SD)		31.54 ± 3.88	46.45 ± 9.03		*Endocrine & Reproductive Medicine*	28 (10.29)	14 (5.11)
**Children**	*None*	183 (67.78)	99 (36.0)		*No specialization*	20 (7.35)	115 (41.97)
	*One or more*	87 (32.22)	176 (64.0)		*Undecided*	96 (35.29)	–
**Training level (trainees)**	*1st year*	26 (9.56)	–	**Authorized training (institution)**	*1 year*	3 (1.10)	0 (0.00)
	*2nd year*	36 (13.24)	–		*2 years*	6 (2.21)	13 (4.71)
	*3rd year*	49 (18.01)	–		*3 years*	2 (0.74)	10 (3.62)
	*4th year*	53 (19.49)	–		*4 years*	5 (1.84)	9 (3.26)
	*5th year*	51 (18.75)	–		*5 years*	23 (8.46)	28 (10.14)
	*5* + *years*	18 (6.62)	–		*Full training authorization*	232 (85.29)	215 (77.90)
	*Specialist*	39 (12.79)	–		*No authorization*	1 (0.37)	1 (0.36)
**Level of care**	*Maximum care*	74 (27.21)	92 (33.45)	**Cancer center certification (institution)**	*Gynacologic Cancer*	20 (7.38)	11 (4.01)
	*University hospital*	127 (46.69)	86 (31.27)		*Breast Cancer*	39 (14.39)	61 (22.26)
	*Office*	6 (2.21)	10 (3.64)		*Breast & Gynecologic*	185 (68.27)	148 (54.01)
	*Other*	2 (0.74)	7 (2.55)		*No certification*	27 (9.96)	54 (19.71)

### Surgery figures of trainees

The trainees performed a median of 125 (IQR: 41–332) non-obstetric surgeries and 75 (IQR: 27–168) obstetric surgeries independently. Detailed figures of the surgeries performed are presented in Table 2. The majority of non-obstetric surgeries performed by the trainees were hysteroscopies and small vaginal procedures, such as dilation & curettage or conisations. Major procedures, such as type-III or type-IV laparoscopy or hysterectomy (open/vaginal), were carried out independently by the trainees to a very limited extent. Breast surgery was also rarely performed independently by trainees.

**Table 2:** Number of independently performed surgeries for the overall trainees (left) and last-year residents (right)

	Overall trainees (*n* = 305)	Last year residents (*n* = 68)
**Non-obstetric procedures**	125 (41–332)	277 (120–453)
**Obstetric procedures**	75 (27–168)	148 (90–243)
**Endoscopic procedures**		
*Diagnostic hysteroscopy*	30 (10–100)	75 (26–150)
*Operative hysteroscopy*	4 (0–15)	7 (1–20)
*Type I laparoscopy (simple complexity)*	10 (2–29)	25 (12–40)
*Type II laparoscopy (medium complexity)*	4 (0–20)	12 (3–31)
*Type III laparoscopy (high complexity)*	0 (0–0)	0 (0–1)
*Type IV laparoscopy (highest complexity)*	0 (0–0)	0 (0–0)
*Urogynaecologic procedure*	0 (0–0)	0 (0–3)
**Open and vaginal procedures**		
*Minor surgery (e.g., conisation/curettage)*	40 (15–100)	85 (40–150)
*Abdominal hysterectomy or adnectomy*	0 (0–3)	2 (0–8)
*Vaginal hysterectomy*	0 (0–2)	0 (0–3)
*Abdominal myomectomy*	0 (0–0)	0 (0–0)
*Radical-oncologic procedure*	0 (0–0)	0 (0–0)
**Breast surgery**		
*Minor breast surgery*	3 (0–10)	5 (2–12)
*Breast conserving surgery*	0 (0–4)	2 (0–7)
*Mastectomy*	0 (0–2)	1 (0–3)
*Sentinel-node biopsy*	0 (0–3)	1 (0–5)
*Axillary lymphonodectomy*	0 (0–0)	0 (0–0)
*Complex-reconstructive procedure*	0 (0–0)	0 (0–0)
**Obstetric procedures**		
*Caesarean section*	60 (25–120)	103 (63–200)
*Vaginal operative delivery*	7 (0–25)	21 (10–40)
*Repair of high-grade birth injury*	0 (0–5)	3 (0–10)

The subgroup analysis of residents with four or more completed years of training showed a median of 277 (IQR: 120–453) performed non-obstetric surgeries and 148 (IQR: 90–243) obstetric procedures. Again, a high proportion of the non-obstetric surgeries were minor procedures such as hysteroscopies, small vaginal procedures, and non-complex laparoscopies. Complex laparoscopies and abdominal procedures are only performed to a very limited extent by last-year residents, as shown in Table 2.

### Logbook

The majority of trainees (65.04%, *n* = 173) and about half of trainers (50.55%, *n* = 139) support that every gynecologic resident should perform 200 minor procedures and 100 major procedures during their residency, which were required in most federal states by the time of the survey. Considerably more trainers (42.91%, *n* = 118) than trainees (28.57%, *n* = 76) stated that the number of required surgeries is too high. Only a few trainees and trainers indicated that the number of required surgeries is too low (6.39%, *n* = 17 of trainees and 6.55%, *n* = 18 of trainers).

A high proportion of trainees (64.79%, *n* = 173), but fewer trainers (39.27%, *n* = 108) reported that the required numbers in the logbook are being rounded up. More than half of the trainees (57.72%, *n* = 157) stated also that the obligatory annual training review meetings, which are documented in the training logbook, do not take place regularly. In contrast, only a minority of trainers (23.19%, *n* = 64) confirmed that annual training review meetings do not take place regularly.

### Evaluation of surgical training

Most participants, 98.19% of trainers (*n* = 270) and 93.38% (*n* = 254) of trainees, stated that surgical training is important or very important. However, trainees rated the quality of surgical training significantly lower than trainers [on a scale from 1 (very good) to 6 (insufficient), mean ± standard deviation: trainees: 3.7 ± 1.4 vs. trainers: 2.7 ± 1.1, *p* < 0.0001].

Regarding the initiation of surgical training, the majority of trainers (65.58%, *n* = 181) reported that training starts at the resident level in their respective institutions, while 24.28% (*n* = 67) reported that it starts in the last year of academic studies. Only a small percentage of trainers (10.15%, *n* = 28) reported that surgical training begins after residency. Trainees stated that they spend an average of 23.94 ± 14.65% of their working time in the operating theatre. With respect to the number of surgeries involving a trainee, 36.43% (*n* = 98) of trainees and 27.53% (*n* = 76) of trainers reported having a resident in the operating theatre in less than half of the surgeries performed.

Trainers and trainees provided contradictory information when asked about the support for surgical training in their institutions. More than half of the trainees (53.16%, *n* = 143) reported that there is no explicit support for surgical training in their institution, but only a few trainers (7.25%, *n* = 20) confirmed the lack of explicit training support. Trainers reported more frequent support opportunities in surgical training, such as internal training courses, time off and budget for external trainings, availability of training models, and regular feedback. Table 3 provides a detailed overview of the support for surgical skills improvement.

**Table 3:** Current support in surgical skills improvement

	Trainees	Trainers
How is surgical training supported in your institution? (Multiple answers possible)		
*Regular internal training courses*	38 (14.13)	187 (67.75)
*Time off for external training*	71 (26.39)	155 (56.16)
*Budget for external training*	92 (34.20)	203 (73.55)
*Training models in institution*	70 (26.02)	139 (50.36)
*Mentoring*	36 (13.38)	91 (32.97)
*Regular Feedback*	34 (12.64)	132 (47.83)
*External job shadowing*	7 (2.60)	112 (40.58)
*No explicit support*	143 (53.16)	20 (7.25)
*Other*	10 (3.72)	15 (5.43)

Trainees identified a lack of structure in surgical training, a high proportion of administrative tasks, a lack of time for teaching in the operating theatre, and limited human resources as the main obstacles for surgical training (Table 4). In contrast, limited time was seen as the main difficulty by trainers. Trainers also identified the complexity of procedures as a main problem for surgical training (Table 4). Neither trainers nor trainees considered motivation a major issue in surgical training. 91.88% (*n* = 249) of the trainees reported that good surgical training would be a reason to change their employer. Concurrently, 55.80% of trainers (*n* = 154) stated that they have problems filling open positions for residents and specialists with surgical experience.

**Table 4:** Current difficulties in surgical training

	Applies completely		Applies predominantly		Applies less		Does not apply	
	*Trainee*	*Trainer*	*Trainee*	*Trainer*	*Trainee*	*Trainer*	*Trainee*	*Trainer*
Limited time resources (tight OR schedule)	126 (46.7)	136 (49.3)	102 (37.8)	95 (34.4)	37 (13.7)	40 (14.5)	5 (1.9)	5 (1.8)
Lacking motivation of trainee or trainer	31 (11.5)	5 (1.8)	100 (37.0)	35 (12.8)	110 (40.7)	126 (46.0)	29 (10.7)	108 (39.4)
Complexity of procedure	55 (20.5)	44 (16.2)	113 (42.0)	136 (50.2)	94 (34.9)	81 (29.9)	7 (2.6)	10 (3.7)
Limited human resources	104 (38.5)	94 (34.8)	94 (34.8)	96 (35.6)	56 (20.7)	61 (22.6)	16 (5.9)	19 (7.0)
Economic pressure	54 (20.2)	57 (21.1)	86 (32.1)	68 (25.2)	92 (34.3)	111 (41.1)	36 (13.4)	34 (12.6)
Administrative tasks (resident)	146 (54.5)	59 (21.5)	83 (30.1)	105 (38.2)	32 (11.9)	95 (34.6)	7 (2.6)	16 (5.8)
Trainer needs procedure for own specialization	56 (20.7)	42 (15.3)	78 (28.9)	69 (25.1)	101 (37.4)	118 (42.9)	35 (13.0)	46 (16.7)
Lack of structure in surgical training	146 (54.1)	39 (14.2)	87 (32.2)	71 (25.9)	32 (11.9)	112 (40.9)	5 (1.9)	52 (19.0)

### Opportunities for improvement

As an opportunity for improving surgical training, a majority of both trainees and trainers indicated that an external higher-level junior fellowship program (mentoring program) would be helpful (trainees: 58.96%, *n* = 158; trainers: 57.04%, *n* = 147). Furthermore, external fellowship programs for specific structured training in oncologic surgery or breast surgery were supported by both trainees (68.23%, *n* = 183) and trainers (67.65%, *n* = 184). A central and regular review of residency training, with potential consequences for the institution in case of non-compliance, was supported by 72.39% of trainees (*n* = 194) and 42.28% of trainers (*n* = 115). Regarding what a trainee can do on an individual level to perform more surgeries and receive better training, the most frequent answers from participating trainers were independent practice (60.36%, *n* = 166), actively asking to operate (58.18%, *n* = 160), increased motivation (53.45%, *n* = 147), accepting overtime (44.72%, *n* = 123), attending external trainings (34.55%, *n* = 95), remaining in the hospital after duty (24.00%, *n* = 66), and participating in internships at external clinics (21.45%, *n* = 59).

## Conclusion

### Principal findings

This comparable large survey, including both OBGYN trainees and trainers, highlights that surgical training in OBGYN is a crucial issue for most participants. However, the quality of surgical training is not rated well, especially by trainees due to the high proportion of administrative tasks and a lack of teaching time in the operating theater. Results from this nationwide survey conducted in Germany show that trainees, if at all, only achieve the required surgical figures in the training logbook for minor surgeries, but not for major surgeries. Trainees report also that the numbers required in training logbooks are often rounded up, and the required annual training reviews do not occur.

### Results in the context of literature

A recent survey of German OBGYN residents revealed a high value placed on learning laparoscopic surgical techniques [8]. However, our study showed that the quality of surgical training in OBGYN was only rated as satisfactory to sufficient, with trainers giving an average rating of 2.7 and trainees giving a rating of 3.7 on a scale from 1 to 6. This finding is in line with previous studies that have evaluated training in OBGYN [5, 7] or in senology [6] and confirms that trainees generally evaluate surgical training more negatively than trainers [5]. Like many other European countries, Germany maintains a logbook to document performed surgical procedures during OBGYN training [3, 4]. Trainers are required to verify the factual numbers of surgeries completed by trainees, but they are not asked to assess the trainees’ surgical skills. At the time of the survey, most German federal states required 100–200 minor surgeries and 50–100 major surgeries during residency training [2].

In our survey, trainees were able to meet the targeted numbers for minor surgical procedures outlined in the logbook. However, there is growing international consensus that more complex surgeries, such as hysterectomies, should also be included in core gynecologic training [4, 8–10]. Our survey revealed that a large proportion of trainees in their final year of training did not achieve the required numbers for these major procedures. This finding is consistent with a German 2011 survey that highlighted a mismatch between actual surgical training and logbook requirements [2, 3]. Notably, international gynecological (oncology) trainees have emphasized the importance of hands-on surgical skills, particularly for complex surgeries and laparoscopies [4, 11, 12]. Our survey supports this view, as trainees reported a preferred minimum of >30 performed laparoscopic surgeries, which is in line with the previous reports [8]. Only half of the trainees in this cohort reported receiving explicit support for their surgical training, highlighting a significant lack of structured educational guidance. A substantial portion of administrative tasks undertaken by trainees and a lack of structure in surgical training were thereby identified as primary obstacles to surgical training, a well-documented issue within the broader context of residency training [13, 14]. Furthermore, trainers underscored the increasing challenge of limited teaching time in the operating room.

Considering the inadequate training situation in structured surgical training for OBGYN residents, discussions about alternative training concepts are warranted. In addition to live training in the operating theatre, simulation-based training has demonstrated enhanced performance among participating residents [15, 16]. These simulations serve as potential additional tools for delivering standardized, in-depth training and should be broadly available in residency training. Nevertheless, one-on-one mentoring by a supervisor remains the core component of surgical training. Only a few study participants in our survey reported having a dedicated mentor or specific supervisor for their surgical training. The lack of mentoring reported by trainees in the survey can be addressed through external mentoring programs, which are widely supported by residents and educators in our study. Recently, fellowship programs established by major German gynecologic societies, such as the Young Academy of Gynecologic Oncology (JAGO) of the North-Eastern German Society of Gynecologic Oncology (NOGGO), the Young Talents of the Working Group Gynecologic Oncology (AGO), and the Junior Academy of the Working Group Urogynecology and Pelvicfloor Reconstruction (AGUB), have been established to provide structured training and mentorship for residents and could address the lack of surgical training during residency, especially for those residents interested in a more comprehensive surgical career in the respective subspecialities. In addition, trainees in this survey demanded a central and regular review of residency training, with potential consequences for the respective institution in case of non-compliance. The “visiting system” established for this purpose by the European Board and College of Obstetrics and Gynecology (EBCOG) and the European Union of Medical Specialists (UEMS) could serve as an example [17]. However, this accreditation program is based on voluntariness and does not include a formal European exam.

## Strengths and limitations

The study’s main limitations are possible selection bias, as it is underrepresented by physicians working in local surgeries and overrepresented by those working in maximum care hospitals. Furthermore, the majority of participants showed a special interest in gynecologic oncology. This selection bias may be attributed to the distribution channels used to disseminate the survey. It is also possible that dissatisfied physicians were overrepresented in the survey, leading to potential biases such as falsely low surgery figures. Another limitation lies in the subjective evaluation of training quality. Incorporating objective metrics or performance outcomes could provide a more comprehensive evaluation of training quality. Additionally, the present study offers a static view of the training situation and no longitudinal data. Longitudinal studies could aid in determining whether the quality of surgical training is progressing, regressing, or staying constant over time… Nonetheless, with 601 participants, this survey is one of the largest and provides the first nationwide data on surgical OBGYN training in Germany, including specific surgery figures for residents. More female participants than male participants are represented, particularly among trainees, which reflects the gender distribution of OBGYN trainee physicians in Germany.

## Implications

In summary, this survey provides an overview of the status of surgical gynecologic training in Germany and emphasizes the need for improvement. To address the unsatisfactory surgical training situation of gynecologic residents, adjustments to the residency curriculum are urgently needed. A structured training program is necessary to provide in-depth surgical training to all residents, especially final-year residents who perform much fewer major procedures than required by the logbook. Independent evaluation mechanisms to track the training progress of each resident, teacher and trainee supervision, and mentoring concepts can aid in implementation. Additionally, fellowship or cross-institutional programs can address the lack of surgical training and provide further opportunities for residents to gain valuable experience. Future studies should address the question of how far structured training programs such as fellowship or mentoring programs can really improve the quality of training in OBGYN.

## Data Availability

The datasets used and/or analyzed during the current study are available from the corresponding author upon reasonable request.

## Abbreviations

AGO: Arbeitsgemeinschaft Gynäkologische Onkologie
AGUB: Arbeitsgemeinschaft für Urogynäkologie und plastische Beckenbodenrekonstruktion e.V
JAGO: Junge Akademie Gynäkologische Onkologie
NOGGO: Nordostdeutsche Gesellschaft für Gynäkologische Onkologie e.V.
OBGYN: Obstetics and Gynecology
